# Horizontal Transfer and the Evolution of Host-Pathogen Interactions

**DOI:** 10.1155/2012/679045

**Published:** 2012-11-26

**Authors:** Elena de la Casa-Esperón

**Affiliations:** ^1^Albacete Science and Technology Park, 02006 Albacete, Spain; ^2^Regional Center for Biomedical Research (CRIB), The University of Castilla-La Mancha, Calle Almansa 14, 02006 Albacete, Spain

## Abstract

Horizontal gene transfer has been long known in viruses and prokaryotes, but its importance in eukaryotes has been only acknowledged recently. Close contact between organisms, as it occurs between pathogens and their hosts, facilitates the occurrence of DNA transfer events. Once inserted in a foreign genome, DNA sequences have sometimes been coopted by pathogens to improve their survival or infectivity, or by hosts to protect themselves against the harm of pathogens. Hence, horizontal transfer constitutes a source of novel sequences that can be adopted to change the host-pathogen interactions. Therefore, horizontal transfer can have an important impact on the coevolution of pathogens and their hosts.

## 1. Introduction

The evolution of pathogens and their hosts is often interpreted as an arms race: while hosts have developed multiple mechanisms to protect themselves, pathogens have generated diverse strategies to evade their hosts' defenses. But pathogens and hosts have also evolved mechanisms that allow a mutualistic coexistence. During their coevolution, a relationship between pathogen and host has been established based on intricate and specialized molecular interactions. One of the possible outcomes of this long-standing and close relationship is the exchange of genetic material. Horizontal or lateral gene transfer (HT) is the nonsexual movement of genetic information between two organisms [1]. These sequences can be modified and adapted (i.e., coopted, domesticated) during the evolution of the recipient species to improve their own survival. When HT occurs between hosts and their pathogens (in one direction or the other), the acquired sequences can be coopted to affect how the two organisms interact with each other.

Pathogens have developed an impressive array of strategies to avoid host defenses, including the interference or disruption of the host defensive mechanisms and signaling cascades. For instance, vertebrate viruses can avoid detection and elimination by the host immune response by obstructing antigen presentation, blocking apoptosis, disrupting complement cascades, and mimicking or modulating cytokines and their receptors, among others [2, 3]. I will discuss how several of these strategies have been achieved through the acquisition and domestication of host genes.

Hosts, in turn, can protect themselves from the deleterious effects of infections by two approaches: resistance and tolerance [4, 5]. Resistant traits reduce damage by limiting the pathogen growth and, therefore, the extent of the infection, sometimes eliminating the pathogen temporarily. Many pathogens evolve very quickly, developing novel or improved infection strategies; this, in turn, has driven a rapid diversification of many host defense proteins [6]. For instance, mitochondrial antiviral signaling proteins (MAVS) have evolved under strong positive selection in primates, driven by viral antagonism [7]. Rapid evolution of host proteins can be particularly important for counteracting molecular mimicry of host molecules, a mechanism developed by some pathogens (usually by coopting horizontally transferred host defense genes) to evade the host protective response [7]. In addition, resistance can also be achieved by hosts acquisition and domestication of genes from their parasites, and several examples will be discussed.

In contrast, tolerance is attained by diminishing the pathogenic consequences of infection without reducing or eliminating the pathogen [4, 5]. Although resistance and tolerance can have similar short-term consequences, they have different long-term dynamics: resistance traits often imply physiological costs for the host; thus, their benefits decrease as the risk of infection diminishes in the population. Therefore, fixation of complete resistance traits is unlikely to occur [4]. In contrast, tolerance does not reduce the infection and represents an advantage as pathogens multiply. Consequently, tolerance traits can spread in the population until they become fixed, resulting in evolutionarily stable associations between hosts and parasites [4]. It has been postulated that mutualism could evolve from parasitism due to natural selection for host tolerance [4]. Moreover, the interaction between the two organisms could be beneficial for both in some circumstances, while disadvantageous to either host or parasite in others [8]. HT can also contribute to this conditional mutualism by providing novel genes to hosts or parasites that allow them to survive without a costly toll on their partners, at least under certain conditions, and several examples will be presented.

**Figure 1:**
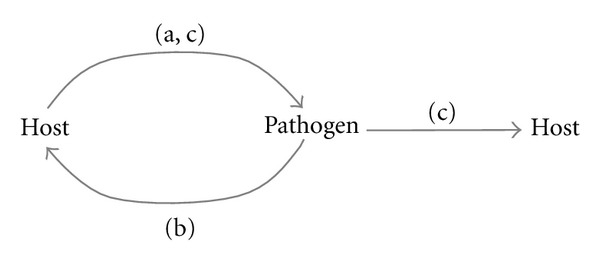
Horizontal transfer of DNA sequences: (a) from host to pathogen; (b) from pathogen to host; (c) from one organism to other through one or more pathogens.

I will start by reviewing the extent of HT in general and the possible role of pathogens as vectors of HT. I will later discuss HT from parasites to hosts and from hosts to parasites, including representative cases of domestication of the acquired sequences (Figure 1). The studies presented here reveal that HT has been a source of novel genetic material that has shaped the interactions (whether purely parasitic or conditional mutualistic) and evolution of many organisms.

## 2. Horizontal Transfer—How Common?

HT is often observed in prokaryotes, as well as in viruses, and has played an important role in their evolution. Through HT, they can incorporate DNA from both related and unrelated organisms and adapt to novel environmental conditions, including the infectious way of life [1, 9, 10]. In fact, HT was first discovered by the ability of bacteria to incorporate drug resistance genes from other organisms; since then, numerous studies have found that horizontally transferred (HTd) sequences constitute a substantial part of the genome of bacteria and archaea, blurring the boundaries between species [11–13]. Prokaryotes cannot only exchange genetic material with other prokaryotes and viruses with viruses, but also between them and with eukaryotes. However, there are few reports of eukaryote-to-prokaryote HT. Prokaryotes (as well as viruses) are unable to process introns, which posses a difficulty for the domestication of many eukaryotic genes [14–16]. It has also been postulated that, being more abundant and diverse than eukaryotes, prokaryotes might have more opportunities for HT and more functionally diverse genes to offer to other organisms [15].

Compared to prokaryotes, HT has been considered a rare event in eukaryotes. An exceptional phenomenon is that of introgression in animals and plants as a result of hybridization between related species: when hybrids survive and reproduce, they may transfer sequences from one species into the other [17, 18]. However, in general, integration of HTd sequences in the germ line can be troublesome in eukaryotes with confined and sheltered reproductive systems [1, 19]. Furthermore, fixation of foreign genes depends on the selective forces operating on the novel acquired sequences and on other factors affecting the population dynamics of each species [15, 20–22]. Nevertheless, there are multiple examples of HT in eukaryotes [1, 19, 20]. Apart from the well-known relocation of genes of endosymbiotic organelles (mitochondria, plastids) to the eukaryotic nucleus, HT is relatively frequent among plant mitochondria and in microbial eukaryotes [1, 20, 23]. In protists, especially in those with phagotrophic lifestyles, HT is quite common, and most transferred sequences are of bacterial origin [20, 24]. This is not surprising since the most common sources of foreign DNA are the organisms in close contact with eukaryotes (particularly bacteria, but also other pathogens and parasites). Bacteria are very diverse and can provide genes for novel functions that might allow eukaryotes to colonize novel environments [15, 20].

In contrast with protists, HT appears to be a rare event in animals and fungi [20, 25–27]. Nevertheless, recent reports have started to unveil the importance of HT in animals. Massive HT has been found in bdelloid rotifers [28]. Studies of transposable elements (which constitute a large part of eukaryotic genomes) have shown that HT has occurred during the evolution of larger animals more often than previously expected [18]. Once transferred, transposable elements can take advantage of their inherent ability to mobilize and integrate into chromosomes, increasing their probability of fixation with respect to other HTd DNA sequences [17, 19, 29]. HT of transposable elements has been observed between closely related species [30–33], but also across distantly related species [34, 35] and even distant locations: such is the case of several transposable element families, which have been found in diverse tetrapods of different continents and even in invertebrates [22, 36–40]. Moreover, the data suggest that each of the transposable element families found in tetrapods have undergone not one but multiple HT events.

## 3. Pathogens as Horizontal Transfer Vectors

Although many studies have shown that HT in vertebrates is more common than previously thought, it is unclear how HT of homologous sequences could occur multiple times in distant species. One of the hypotheses is that pathogens might act as vectors capable of capturing host sequences and donating them to other hosts [17, 19]. This hypothesis is supported by several observations [41]; for instance, several of the transposable elements found in tetrapods have also been observed in several parasites [37, 39], such as trypanosomes. These parasitic protozoa cannot only capture but also donate sequences to their hosts: HT of trypanosome sequences has been reported in infected human beings, who vertically transmitted them to their children [42]. In the genus *Drosophila*, a parasitic mite appears to be responsible for the HT of P elements among different species [43]. Pathogens such as bacteria and viruses could also play a key role as HT vectors; for instance, *Wolbachia* (an intracellular parasitic bacteria that is horizontally transmitted) can donate genetic material to the nuclear genome of its insect and nematode hosts [44, 45]. Moreover, *Wolbachia* can be infected by bacteriophages, and it has been suggested that these viruses might mediate HT among intracellular bacteria [17]. Viruses might also mediate the HT of transposable elements from lepidopteran hosts to their parasitic wasps [19, 46].

In fact, viruses are potential HT vectors not only among vertebrates, but also in other eukaryotes and prokaryotes. The genomes of large DNA viruses, such as poxviruses, contain many genes derived from their bacterial and eukaryotic hosts [9, 10] (including transposable elements [47]). Poxviruses also seem to have changed hosts recurrently during their evolution [48], broadening their opportunities to transfer sequences among distantly related species. For instance, a transposable element related to snake sequences has been found in the genome of a taterapox virus isolated from a rodent [49]. Therefore, poxviruses may be good HT vectors due to their large genomes and low host and cell specificity. Other potential vectors are double-stranded RNA viruses, which are likely the donors of multiple sequences found in the nuclear genome of very diverse eukaryotes, including plants, fungi, protozoa, arthropods, and nematodes [50–52].

Regardless of the frequency of HT events in diverse species, the fact is that many of them are deleterious, are not transmitted, or do not succeed in the populations. [12]. So why have HTd sequences been retained in some populations throughout the evolution? Transposable elements are unique because of their intrinsic ability of propagating. But regardless of their mobile or nonmobile nature, horizontally transferred sequences can carry new coding or regulatory sequences to the recipient organisms, upon which natural selection can operate [53]. Therefore, HTd sequences have the potential of providing novel functions (or improved versions of existing ones) that turn out to be beneficial for the receivers. Indeed, HTd sequences have at times been domesticated (for instance, for protective or pathogenic functions in the case of hosts and parasites) and, thus, successfully maintained in the genome of the recipient species.

## 4. From Host to Pathogen: Horizontal Transfer of Genes That Modulate the Immune Response

Pathogens can harbor two particular classes of HTd genes in their genome. The first class includes HTd genes that were acquired from other pathogens and provided functions related to infectivity. Such is the case of the transfer of fungal genes, or even entire extranumerary chromosomes, that converted nonpathogenic fungi into pathogens [27, 54]. The HTd genes that contributed to the adaptation of nematodes to plant parasitism are another example [35, 55]. The second class of HTd genes includes those that were acquired from the host. Physical proximity obviously facilitates HT, although it does not guarantee that the inserted sequence will be retained in the population. But modifications of host genes can provide selective advantages and drive their fixation in the pathogen population, especially when such adaptations serve to elude or counteract the host defenses.

Examples of host-to-pathogen HT are found in very diverse species. For instance, there are several reports of bacterial sequences of eukaryotic origin [56–58]. Some of these sequences might have been coopted by bacteria to their advantage against their host. As previously discussed, several parasitic animals contain transposable elements likely of host origin [41, 43]. HT from host to parasitic plants can also occur, affecting not only mitochondrial genes, but also nuclear sequences [59–61]. For instance, the parasitic flowering plant *Rafflesia cantleyi* expresses several genes (mostly nuclear) that were likely acquired from their hosts, which are also flowering plants. In addition, many vertically inherited genes in the *Rafflesia* genome display codon-usage properties that are more similar to those of the host genes than to sequences of related species [61]. Therefore, while the close host-parasite relationship could facilitate HT, maintenance of the transferred genes may have been favored by codon-usage convergence between *Rafflesia* and its host.

Viruses are opportunistic and frequent receptors of host genes. Among animal viruses, HT of host genes has been particularly common in nucleocytoplasmic large DNA viruses, such as poxviruses and herpesviruses [2, 3]. These viruses are large enough to allow the incorporation of foreign genes. However, the number and size of the insertions are limited by the packaging restrictions of these viruses; in addition, HTd sequences can be removed over time by deletions (which occur at a high rate in viruses). Nevertheless, large DNA viruses have been quite proficient at domesticating host genes for eluding multiple aspects of the immune response [2, 62–65].

Poxviruses are particularly prone to exchange genes with their hosts [9, 14, 47, 48, 66]. Within this viral group (which includes the vaccinia and smallpox viruses), many of the host-derived genes vary among different genera. This suggests that HT may have contributed to poxvirus diversification and adaptation to diverse hosts [14, 65]. Host-derived genes tend to be located at the two ends of the poxviral linear genome, along with poxviral genes involved in limiting the host antiviral response; these extremes seem more recombinogenic than the central region, which contains conserved and essential genes [14, 47]. In an extensive analysis, Hughes and Friedman [9] characterized 16 poxviral gene families that were acquired from their hosts. These findings have been further expanded in other studies [66–69], supporting the existence of many host homologues in the genomes of poxviruses. Other studies have also reported numerous HT events from hosts to herpesviruses during evolution [2, 70–73].

The genomes of poxviruses and herpesviruses contain several host homologues coding for proteins that modulate the immune response. Many of the coopted HTd genes code for proteins that interact with host ligands or receptors, targeting cytokines, and innate immune mechanisms [3, 63–65]. For instance, homologues of chemokines and chemokine receptors are found in both poxviruses and herpesviruses [2, 62, 73]. The host versions of these proteins recruit leukocytes to the sites of infection and, thus, are key components of the antiviral defense pathways. To oppose their effects, viruses express proteins that mimic these components and interfere with their normal functions. One of the best-studied examples is the HT of mammalian interleukin-10 family members (*IL-19, IL-20,* and *IL-24*) to viruses. In human Epstein-Barr virus (a herpesvirus), a viral homologue was identified as an agonist with impaired binding to the host *IL-10* receptor [74]; moreover, it contributes to the downregulation of the host immune response during early infection [75, 76]. Homologues of *IL-10* family members have also been found in other herpesviruses (such as the cytomegalovirus [77]), as well as in poxviruses. Studies in the latter have shown that *IL-10* family homologues also have immunosuppressive properties, because they contribute to delaying the development of acquired immunity to *Orf virus* in humans and to vaccinia virus in mice [78, 79]. In addition, phylogenetic studies have shown that *IL-10* homologues were acquired multiple times in poxviruses by independent HT events [48, 66, 80]. The recurrent HT of these host genes to poxviruses and herpesviruses further supports the idea that the acquisition of *IL-10* family members provided a selective advantage to them [80].

Other examples of genes of the host defense machinery that have been horizontally transferred to poxviruses include those coding for proteins of the MHC class I, other interleukins and their receptors and the interferon gamma receptor [9, 65]. Although in some cases they interfere with the normal action of homologous proteins in the host, in other cases the acquired genes have adopted new immunomodulatory functions [65, 81]. Poxviral genomes have also incorporated host sequences for serine protease inhibitors (serpins, involved in inflammation control), glutaredoxin (which has antiapoptotic effects under oxidative stress), and glutathione peroxidase, [9, 14, 66]. In the host, glutathione peroxidase has a protective role against oxidative stress, a function that has been adapted by certain poxviruses to protect themselves and the infected cells from the oxidative damage that results from the immune response [82].

In all these examples, host-to-virus HTd genes enhance viral survival and propagation by interfering with the immune response; however, other host-derived genes adopted by viruses contribute to control their own infectivity. Such is the case of the interleukin-24 (*IL-24*) gene homolog found in the Yaba-like disease virus (a poxvirus), which appears to reduce its virulence upon infection [83]. Another example found in poxviruses is that of homologues of the mammalian *Schlafen* gene family. These genes are expressed in cells of the host immune system and were horizontally transferred to orthopoxviruses, probably from rodents [67, 84]. Studies of the *Schlafen* viral homologue in a recombinant viral model suggested that the gene product might contribute to decrease virulence [85]. Although little is known about the function of the *Schlafen* genes in the host, functional studies of viral *Schlafen* copies and of the interactions between the host and viral homologues may be instrumental for unveiling the contribution of this gene family to the immune response [84]. Similarly, studies of other HTd genes in both hosts and pathogens might provide complementary insights into diverse aspects of immunology and infectivity. In addition, HTd genes involved in modulating immunity and virulence constitute potential therapeutic targets [3, 64, 65, 86, 87].

The existence of host-to-virus HTd genes that restrain their infectivity supports the notion that the evolution of hosts and viruses, like other organisms considered pathogens, cannot simply be viewed as an arms race [8]. For example, upon infection, herpesviruses remain usually latent during the host lifetime and only become reactivated and pathogenic under particular conditions. Coevolution with their hosts and their immune response has allowed these viruses to persist with little cost to animals. In the course of this evolution, herpesviruses have horizontally acquired certain vertebrate genes and coopted them to evade the immune response [73, 75, 76]. Moreover, during latency, herpesviruses can also modulate the defenses against other diseases, providing immunity against some secondary infections in mice [88, 89]. Therefore, herpesviruses could be considered as part of our “normal” defensive response, with both beneficial and detrimental consequences [89]. Through evolution, they have acquired some advantageous sequences from their hosts, but they have also evolved mechanisms to modulate the immune system that benefit both parts.

In summary, HTd genes from host to pathogens can be “domesticated” by the latter to regulate not only the host defense response, but also their infectivity. Consequently, these genes have contributed to the coevolution between pathogens and their hosts. On one hand, HT provides new tools to the pathogen to ensure its propagation and even to occupy new niches. On the other, genes that originally contributed to the host defensive response may become instruments to elude it, leaving the host exposed to the pathogen attack. Thus, it has been postulated that HT of host defense genes to viruses and other pathogens may have been counteracted through rapid evolution of the original copies in the host [6]. The ability of pathogens to exploit the similarities of their gene products with their host homologues to elude the defense response may have contributed to drive the rapid diversification of host defense genes in mammals [6].

## 5. From Pathogen to Host: Horizontal Transfer Can Shape the Evolution of the Host Defense Mechanisms

Recently several reports have brought light into the relevance of HT from pathogens to their hosts, even to those species with a sheltered germ line (such as vertebrates) [19]. As previously discussed, there are several reports of animals bearing certain sequences that are more similar to sequences of their parasites than to those of related species; these sequences were likely transferred from the parasites to their hosts. The sequence donors include eukaryotic parasites (such as trypanosomes [42, 43]), prokaryotic pathogens (such as *Wolbachia* [44, 45]), and viruses (discussed below). In plants, several data support the existence of HT of mitochondrial genes from parasitic to host flowering plants as well as HT of genes of pathogenic bacteria to their host plants [90, 91].

In some cases, the sequences acquired by HT have been modified, adopting novel functions in the recipient hosts. Of particular interest are those genes that hosts have coopted to resist pathogens. Most of the known examples are found in viruses and the organisms they infect. In fact, several studies of coevolution of diverse viruses and their hosts have arrived to the same conclusion: viral sequences have been the likely origin of many host defense mechanisms (reviewed in [92]).

This is not surprising if we consider that some viruses can provide resistance to their prokaryote or eukaryote hosts against secondary infections [88, 92]. For instance, bacteriophages often bear antiviral defense systems. The intimate relationship between phages and bacteria facilitates HT, and the advantageous nature of antiviral sequences promotes their retention and selection in the host. An example is found in restriction-modification systems present in prokaryotes as major defenses against viral attacks. Some of them have also been found in prophages, suggesting a viral origin of the restriction-modification genes [92, 93]. In addition, bacteria and archaea also have virus resistance mechanisms based on the incorporation of viral sequences: the clusters of regularly interspaced palindromic repeats (CRISPs). These loci integrate small segments of bacteriophage and plasmid DNA to generate RNA-mediated defenses against viral and plasmidic invasions [92, 94, 95].

HT of viral genes can also contribute to eukaryotic mechanisms of resistance to pathogens. It has been proposed that the widespread HT of double-stranded RNA viral sequences to several eukaryotic groups generated novel functional genes, some of which could be involved in protection against exogenous viral infections [50–52]. In many eukaryotes, an antiviral defensive response with possible viral origin is that provided by small RNAs, which interfere or silence viral gene expression [96]. Studies of *C. elegans* small RNAs suggest they were originated from transposable elements and endogenous retroviral sequences [92].

In vertebrates, retroviruses constitute a major source of exogenous sequences (up to 8% of the human genome [97]), because they can integrate into their hosts genomes as proviruses, occasionally, reach the germline of the infected animals, and get passed on to the next generations. Consequently, retroviral sequences, as well as DNA from other endogenous viral elements, have been coopted numerous times by the hosts. In some cases, they have been domesticated to interfere with molecular interactions required for virus integrity and infectivity, including viral-host interfaces. The best-known example is the *Friend-virus-susceptibility-1* gene (*Fv1*), a retrovirus-resistance gene found in mice. It is a domesticated version of the *gag* gene of the MuERV-L retrovirus [98–100]. Other retroviral genes coopted by their hosts to serve as antiviral defenses include *Fv4*, *Rcmf,* and *Rmcf2*. All these genes are derived from the *env* genes of endogenous retroviruses and are found in mice, in which they interfere with the activity of viral receptors and with viral entry [99, 101]. A different example of antiviral sequence is *Apobec3*. There are several alleles of this gene in mice, and the allele that provides the most resistance has the highest levels of expression. It turns out that this elevated expression is due to the intronic insertion of a transcriptional enhancer of retroviral origin [99, 102]. Therefore, not only viral genes, but also regulatory sequences can be coopted by hosts for antiviral purposes.

At a larger scale, Villarreal [92, 103] has proposed that, not only individual genes, but also coordinated processes coded by multiple genes in viruses have been the likely origin of the vertebrate adaptive immune system. This hypothesis is based on several observations: first, the emergence of a complex immune system in jawed vertebrates; second, the large viral colonization and genome expansion occurred in teleost fish, compared with those of their presumed ancestors; third, the presence in viruses of elements or networks that resemble several components of the adaptive immune response (for instance, T-cell receptors have several features that are similar to those of viral receptors) [92, 103, 104]. Therefore, HT and domestication of viral sequences could have originated the basal components of a highly complex adaptive immune system in the emergent vertebrates.

## 6. Conclusions

The tight physical and evolutionary relationship between pathogens and their hosts facilitates HT of DNA sequences. Although the acquisition of host genes by viruses, prokaryotes, and other parasites has been recognized for a long time, the relevance of HT in the opposite direction, from pathogens to their hosts (and especially to those that seemed more protected from germ line insertions, such as vertebrates), has been only acknowledged recently. However, one thing is the transfer of a DNA sequence and another is its spread and maintenance in the population, which depend on the selective forces operating on the foreign sequence. Any HTd sequence that provides a selective advantage to the host against the pathogen or *vice versa,* or is able to benefit the coexistence of both, is more likely to be retained than a sequence that does not provide any competitive advantage. HTd genes have been coopted by pathogens to modulate the host defense response and ensure their survival and multiplication, and have been domesticated by hosts to improve their protection against deleterious infections. In addition, pathogens may serve as HT vectors between hosts. Therefore, horizontal gene transfer can be an important factor in the evolution of hosts, pathogens, and the ways they interact with each other.
